# A Novel Hydraulic Actuation System Utilizing Magnetorheological Fluids for Single-Port Laparoscopic Surgery Applications

**DOI:** 10.3390/ma13061380

**Published:** 2020-03-20

**Authors:** Ali K. El Wahed

**Affiliations:** Mechanical Engineering, University of Dundee, Dundee DD1 4HN, UK; a.elwahed@dundee.ac.uk; Tel.: +44-1382384496

**Keywords:** smart materials, magnetic-responsive materials, magnetorheological fluids, smart material actuator, smart hydraulic actuator, single-port laparoscopic surgery (SLS)

## Abstract

Single-port laparoscopic surgery (SLS), which utilizes one major incision, can deliver favorable cosmetic outcomes with fewer patient hospitalization stays and less postoperative pain. However, current SLS instruments, which are rigid and slender, have been suffering from several drawbacks, including their inability to provide the optimum articulation required to complete certain SLS tasks. This paper reports on the development of a lightweight smart hydraulic actuation system that is proposed to be embedded at selected joints along current SLS instruments, in order to enhance their adaptability with a higher level of stiffness and degrees-of-freedom. The developed smart actuation system utilizes both conventional hydraulic and magnetorheological (MR) fluid actuation technologies. Electromagnetic finite element analyses were conducted to design the electromagnetic circuit of the smart actuator. A prototype of the developed actuation system was manufactured, and its performance was assessed using a dedicated experimental arrangement, which was found to agree well with the results obtained using a Bingham plastic theoretical model. Finally, the present design of the developed smart actuation system permits an angulation of about 90° and a maximum force output in excess of 100 N, generated under a magnetic excitation of about 1.2 Tesla, which should be sufficient to resist torques of up to 500 mNm.

## 1. Introduction

Single-port laparoscopic surgery (SLS) is a new paradigm in minimal-access surgery, which aims to improve cosmesis and reduce complications such as ports site hernias, haematomas and wound infections associated with the standard multiport laparoscopic approach. In order to advance the safe application of the SLS procedure, however, several technical challenges still need to be addressed, which are mainly in the area of instrumentation. Most of the current difficulties are attributed to the fact that in SLS, several rigid tools penetrate through a single trocar port, which interact with low stiffness organs that may move or deform. As a result, inadequate exposure of the surgical field, a lack of assistants for organ retraction, instruments crowding due to limited angulations and a restricted range of movements are some of the real issues which impede the dissemination of SLS [1]. These impeding factors potentially increase the operative time of SLS procedures, and have bearings on the safety of patients—especially during the learning curve experienced by surgeons. Several technological advancements have been proposed to overcome some of the above issues, which include specially designed ports to accommodate multiple instruments through a small incision [2], an oblique viewing camera with a flexible tip [3], right-angled light cables for cameras [4] and pre-bent instruments [5]. However, some comparative studies have reported conflicting results in the application of pre-bent and flexible SLS instruments [6].

Recently, there have been attempts to develop a new range of SLS instruments with onboard actuators, with the aim to improve the efficiency and safety of SLS procedures. However, the imposed dimension restriction on SLS instruments—due to the size of the access ports—has narrowed the range of the actuators that could be considered for SLS applications. As a result, the size of the actuator and its performance, including its angulation range and force/torque output, are considered the most important design criteria that are taken into account when SLS articulating instruments are developed. Early attempts of SLS instrument actuation, using pneumatic actuators and linear electric motors [7,8], for example, were hampered, as it was obvious that their miniaturisation to permit their insertion into narrow surgical trocars severely reduced their output capability. In addition, these actuation systems usually require multiple cables and tubes, which make SLS instruments bulky, heavy and mechanically complex surgical systems. These issues have made it difficult for current systems to find their successful deployment in most SLS theatres. Consequently, novel, compact and lightweight actuation systems with adequate mechanical simplicity and robustness must be developed in order to revolutionise the function of current SLS tools [9].

Magnetorheological (MR) fluids, which belong to the general area of smart materials, can exhibit massive and reversible changes in their rheological properties such as yield stress, when subjected to external magnetic fields. These fluids involve the suspension of solid magnetisable particulates—typically micrometres in size—in nonmagnetic liquid carriers. A typical MR fluid is made of carbonyl iron particles suspended in hydrocarbon oil [10]. The fluid response to an applied magnetic field is the familiar chaining of the particulates in the direction of the applied field, and the resulting “solidification” or increase in their yield stress. The efficiency of MR fluids is usually assessed through the fluid yield strength in response to the applied field. Yield stresses of close to 100 kPa were reported under a magnetic field strength of about 1 Tesla [11]. The response time of MR fluids is highly dependent on the rising time of the magnetic field, which is proportional to the inductance and resistance of the energising electromagnetic coil. The fast, strong and reversible gelation provides a novel and efficient way to transfer energy and control motion. Although MR fluids were discovered in the forties of the last century [12], they did not find their routes into real industrial applications for several decades, which was mainly due to the fact that early MR fluids were not stable. However, recent advances in the mechanical, electrical and chemical characteristics of these fluids have permitted the development of novel electromechanical devices, which have been successfully utilised to optimise the function of many commercial automotive, aerospace and structural systems. The majority of these devices have employed the fluids in a simple flow [13,14] or shear mode [15,16] of operation, where the fluid flow is controlled in the former, while the fluid shearing is controlled in the latter. Furthermore, an alternative arrangement, squeeze mode, in which the fluid is subjected to compressive and tensile stresses, has been identified for small motion isolation [17,18]. In addition, it has been recently reported that the efficacy of MR fluid devices could be enhanced under a mixed-mode operation [19,20]. The comparative performance of MR fluid devices using theoretical and experimental procedures has been extensively reported in the technical literature [15,21,22]. A similar approach is adopted in the current investigation, when the performance of the developed MR device was assessed experimentally, which was compared with the results obtained using a Bingham plastic theoretical model.

This paper reports on the development of a smart actuation system that utilises hydraulic power and MR fluid technologies for actuation and position locking, respectively, which is designed for SLS instruments to provide them with the ability to reconfigure and stiffen, in order to achieve the optimum angulation required for most SLS tasks. In this study, the performance of the new actuator under various input conditions was assessed using theoretical and experimental approaches.

## 2. Materials and Methods

### 2.1. Design Concept of a Smart Hydraulic Actuation System for Single-Port Laparoscopic Surgery (SLS) Applications

**Figure 1:**
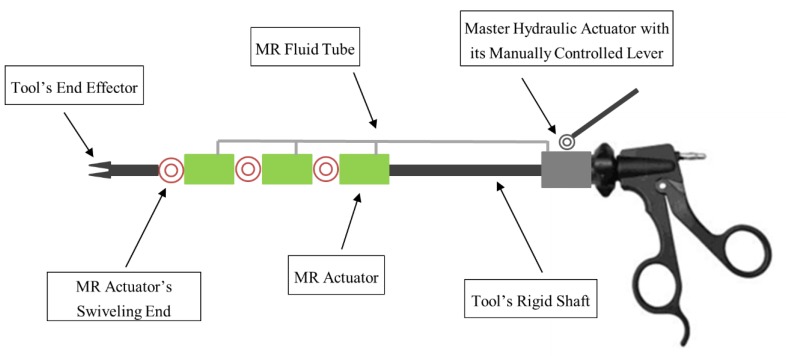
Schematic diagram of the proposed smart actuation system with its three MR actuators, single hydraulic actuator and single fluid delivery tube.

A magnetorheological (MR) fluid-based hydraulic actuation system was proposed and investigated in this work, which aimed to improve the function of SLS instruments and enable their smart articulation. It has been reported in the literature [23] that the exploitation of conventional hydraulic actuators for miniature-size applications has been hindered by their relatively bulky components, including the multiple onboard connecting tubing system and complex control strategies. Therefore, the current hydraulic actuators, which utilize high-precision mechanical fluid valves, are too large to be directly integrated onboard SLS instruments and safely pass through a single trocar port. In contrast, MR actuators could be designed to have a relatively simple structure, which could be miniaturized to a compact size. Additionally, a number of these could be embedded at selected joints along SLS instruments, as part of a hydraulic system, to provide them with adaptability—a role that would enable them to change their stiffness and degrees-of-freedom, and hence transform them into articulated structures without compromising patients’ safety. The proposed smart MR hydraulic actuation system, which is shown in Figure 1, is designed to allow MR devices working in the valve mode to act as slave actuators, responding to the pressure provided through a single fluid delivery tube by a single master hydraulic actuator, which could be mechanically manipulated. As can be seen, three MR slave actuators are proposed to be imbedded onboard the SLS tool shaft, which should provide the tool with six degrees of freedom, including those provided by the main shaft of the tool.

The proposed smart hydraulic actuation system eliminates the requirement for the external fluid reservoir and complex control unit, which are typical of a conventional hydraulic actuation system. In the smart actuation system, the master hydraulic actuator is designed to be manually controlled by the surgeon, which provides the required pressure to initiate the actuation of the MR slave devices. Furthermore, each of the MR actuators is designed to be equipped with an individual swiveling joint, which could transfer their linear stroke into the required angulation of the SLS instrument. In addition, the design permits the MR actuators to be individually controlled, which could result in the tool’s angulation in three different planes. In particular, when an angulation in a certain plane of the SLS instrument is in demand, the corresponding MR actuator is opened while the rest of the MR devices remain closed. The surgeon then manually controls the actuation stroke of the master hydraulic actuator with its lever mechanism, which should transmit the hydraulic pressure to the opened slave actuator, and the corresponding joint should start swiveling accordingly. The angulation of the deforming joints could be designed to be proportional to the angulation of the rotational lever, which provides the surgeon with direct feedback of the posture of the SLS instrument while it is inside the patient. Once the desired angulation of the SLS instrument is achieved, all the MR actuators are activated, and accordingly the posture of the surgical instrument is locked along the required shape. As a result, the control strategy of the proposed smart actuation system is extremely simplified. In addition, compared to complicated conventional mechanical valves, the simple structure of MR actuators can practically increase the operational life and lower the cost of the hydraulic actuation system.

### 2.2. MR Fluid Actuator Design

**Figure 2:**
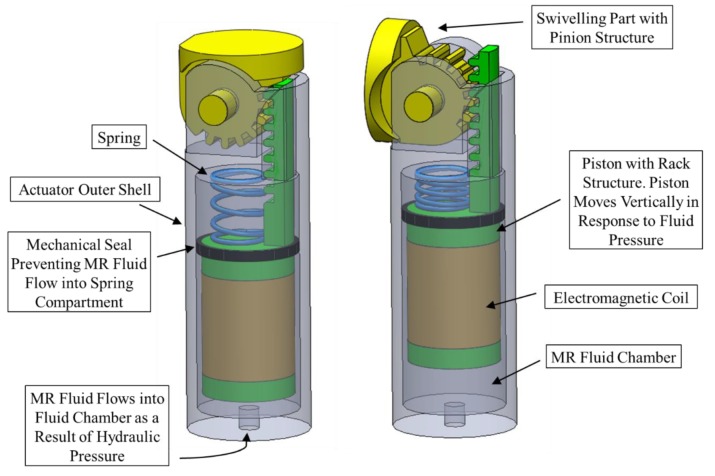
MR fluid actuator with its rack and pinion mechanism shown in its straight (left) and bent positions (right).

Since the proposed MR fluid actuators are aimed to be embedded onboard SLS instruments, which are supposed to pass through surgical trocars where the size is generally limited to a maximum of 15mm in diameter, they were designed with a cylindrical shape and an external diameter of 12 mm. The MR actuator, which is shown in Figure 2, was designed to work under a shear mode that permits the actuation of its piston in response to the MR fluid pressure supplied by the master hydraulic actuator.

The electromagnetic coil is accommodated inside a recess along the centre part of the piston, which is equipped with a seal to prevent the flow of the MR fluid into the upper chamber of the actuator. Furthermore, the stroke of the actuator was designed to enable the achievement of a target angulation of about 90°, whilst its overall length was limited to 27 mm, in order to allow at least three MR actuators to be embedded along the shaft of an SLS tool.

The actuator was designed so that the linear motion of its piston is converted into a rotational motion, which is required to cause the bending of the surgical instrument by using a rack and pinion structure joint. This mechanism is again shown in Figure 2, where the new actuator is illustrated in its straight and bending positions. The same mechanism is proposed for the master hydraulic actuator, with the swivelling component connected to an extended lever, which the surgeon could rotate to generate the required pressure for the actuation of the slave MR devices. When the required bending angles of the slave actuators are achieved, the MR devices are activated. This causes the MR fluid, which is sandwiched between the piston and the shell of the actuator, to solidify under the effects of the passing electromagnetic field generated by the electromagnetic coil element, and creates the required mechanical locking action to maintain the tool along the intended bent posture. If a different bending of the tool is required, the surgeon first deactivates the MR devices before manipulating the lever of the master actuator in the required direction, after which the MR devices are activated again. Of course, the straight posture of the tool could be regained following the same procedure, which is also aided by the equipped compression springs. As is the case with other MR fluid actuators, the locking mechanism strength provided by the current design is proportional to the strength of the applied magnetic field.

MR fluid gaps of between 0.5 and 2.0 mm have been reported in the literature [24]. In the present investigation, however, the MR fluid gap size was assumed to be 0.5 mm. This small gap size was necessary in the current design of the MR actuator, due to the imposed limitation on the overall diameter of the actuator. It was also decided to employ a commercial MR fluid type Lord MRF241-ES (Lord Corporation, Cary, NC, USA) in this investigation, which has a viscosity (at 25 °C) *η* of 88 mPa-s, and is capable of providing yield stresses in excess of 67 kPa when energized by magnetic field intensities in the region of 200 kA/m [25].

**Figure 3:**
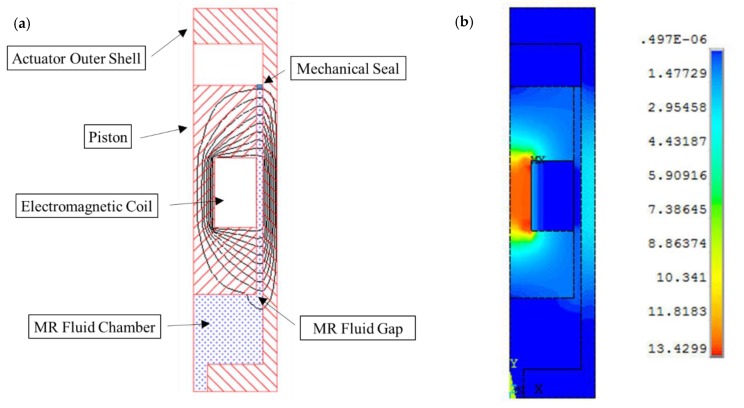
Half-section views of the MR actuator showing contour plots of the magnetic flux lines (**a**) and the magnetic flux density distribution (**b**) in the actuator body (current density = 16,000 kA/m^2^).

Electromagnetic finite element analyses (FEA) were carried out using ANSYS software (Version 14, ANSYS Inc., South pointe, OH, USA) to design the electromagnetic circuit of the MR actuator, which is necessary to generate the magnetic field required to energize the fluid in the gap between the piston and the shell of the actuator. The ANSYS high-accuracy electromagnetic element type “Plane 53” was used in the present simulation. Plane 53 is a quadratic element that is defined by eight nodes, with up to four degrees of freedom per node [26]. It is also suitable to model 2-D axisymmetric magnetic fields, which is the option required in the present actuator device design. In this investigation, a trial and error technique was applied to determine the optimum mesh for the magnetic simulation, which resulted in meshed areas with quad-shaped elements and a minimum element edge length of 5 × 10^−4^ m. The material properties were defined by assigning each material a constant relative permeability when values of 10,000, 1 and 5 were assumed for the magnetic, nonmagnetic and MR fluid materials, respectively. An important step in this simulation involved the application of the loads to which the model was subjected. This included the setting of the current density of the electromagnetic coil to the required value, which was dependent on several parameters, including the wire diameter and the applied current. In this investigation, a self-fluxing polyurethane-coated solid copper wire with a diameter of 0.25 mm was selected for the coil, while the current was changed to between 0.2 and 1.6 A. The electromagnetic model was then solved, and the results were obtained. Figure 3a,b show the contour plots of the magnetic flux lines and the magnetic flux density distribution, respectively, in a half-section of the MR actuator when a current density of 16,000 kA/m^2^ was assumed.

**Figure 4:**
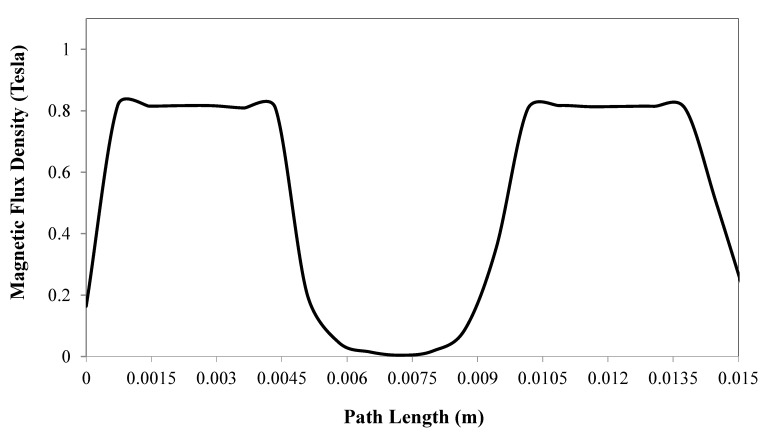
Magnetic flux density variation along a median path inside the MR fluid gap between the piston and the shell of the actuator (current density = 16,000 kA/m^2^).

Figure 4 shows the magnetic flux density variation along an imaginary pathway that is assumed in the middle of the MR fluid gap, which starts from a level parallel to the top end of the piston and ends at a point that is level with the lower end of the piston. The results in this figure (obtained for the same current density of 16,000 kA/m^2^) show that the magnetic flux drops sharply within the centre part of the fluid volume that is opposite the electromagnetic coil element, which is an issue that was also encountered with the development of other MR fluid devices [27,28,29].

## 3. Results and Discussion

This investigation has considered initial steps towards the practical development of a smart hydraulic actuation system for single-port laparoscopic surgery (SLS) applications, which comprise a single master hydraulic actuator and three onboard slave magnetorheological (MR) fluid actuators. In particular, the current work is focused on the design and characterization of the smart system, with its MR fluid actuators being proposed to be embedded onto the shaft of a conventional SLS instrument to allow it to produce the required SLS angulation in response to simple manipulations of the master hydraulic actuator carried out by the surgeon. When activated, the developed MR actuators should provide sufficient locking forces, through which the posture of the SLS tool should be maintained during SLS procedures. Since the designed MR actuator works under the shear mode, the total locking force (*F_t_*) provided by the MR damper is equivalent to the sum of the magnetic field-dependent damping force (*F_τ_*), the viscous damping force (*F_v_*) and the friction force (*F_f_*) [30,31].

(1) $$F_{t}=F_{\tau }+F_{v}+F_{f}$$

According to the parallel-plate Bingham model [32], *F_τ_* is given by

(2) $$F_{t}=\left(2.07+\frac{12Q\eta }{12Q\eta +0.4wh^{2}\tau_{y}}\right)\times \frac{\tau_{y}L_{e}A_{p}}{h}\times \sin v$$

where *Q* is the volumetric flowrate, *η* is the apparent viscosity at zero magnetic field, *τ_y_* is the field-dependent dynamic yield stress, *w* is the mean circumference of the shear gap, *h* is the shear fluid gap, *L_e_* is the effective axial pole length, *A_p_* is the piston area and *v* is the velocity of the piston.

The viscous damping force (*F_v_*) is provided by the following equation [32]

(3) $$F_{v}=\left(1+\frac{whv}{2Q}\right)\times \frac{12\eta QL_{p}A_{p}}{wh^{3}}$$

where *L_p_* is the length of the piston.

**Figure 5:**
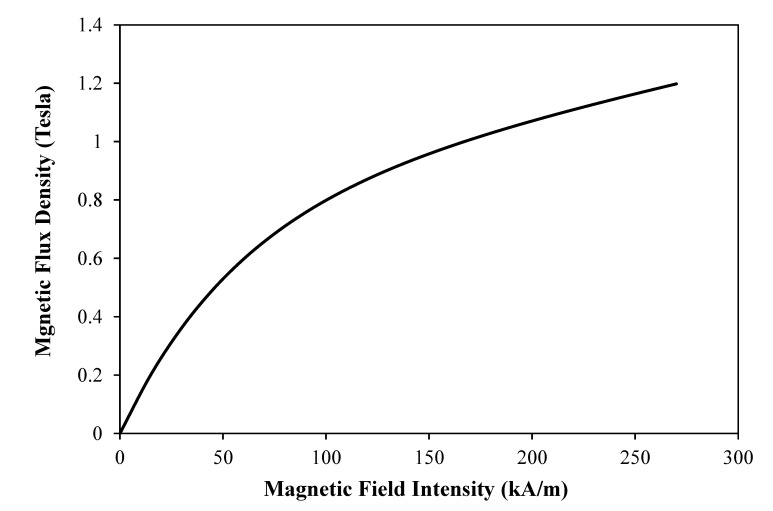
Magnetic flux density versus magnetic field intensity for Lord MRF241-ES fluid.

The MR fluid yield stress (*τy*) was calculated as a function of the particle volume fraction (*ψ*), as well as the magnetic field intensity (*H*), using the following empirical equation [33]:

(4) $$\tau_{y}\;\left(H\right)=C\times \;2.717\times \;10^{5}\times \Psi^{1.5239}\times Tanh\;\left(6.33\;\times \;10^{-6}H\right)\;$$

where *C* is a constant that is set to 1.1253 for the employed water-based MR fluid, while *Ψ* is set to 41%. The relationship between the magnetic flux density (*B*) and the magnetic field intensity (*H*) for the employed fluid was estimated using the following empirical equation [33]:

(5) $$B=1.91\times \Psi^{1.133}\times \left[1-Exp\;\left(-10.97\;\times \;\mu \times H\right)+\mu \;H\right]$$

where *μ* is a magnetic constant which is set to 1.566 × 10^−6^. Figure 5 shows the relationship between the magnetic flux density and the magnetic field intensity of the employed MR fluid.

The important design parameters of the developed MR actuator, including the diameter of the piston (*D_p_*), the piston height (*L*), the effective axial pole length (*L_e_*) and the fluid shear gap (*h*), are shown in Table 1.

**Figure 6:**
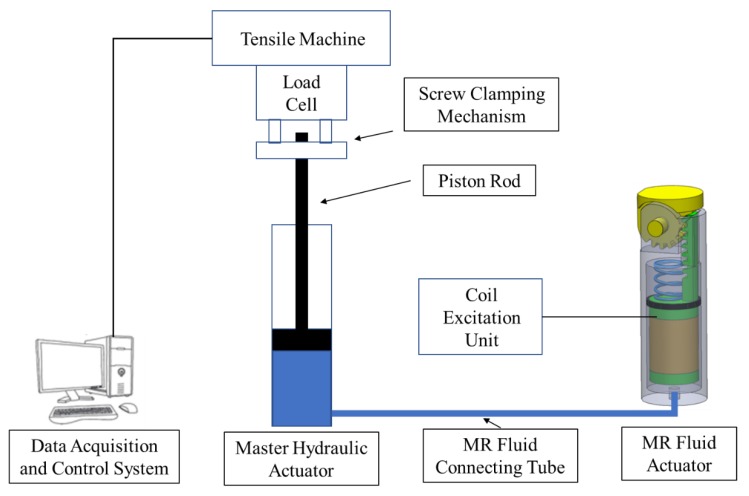
Schematic diagram of the experimental setup for testing the smart hydraulic actuation system.

The new actuator was manufactured using a conventional Computer Numerical Control (CNC) machining facility. The machined magnetic parts were subsequently annealed to allow them to regain their original magnetic properties, as well as to remove any carbon residue from their surfaces. An experimental facility, which is shown in Figure 6, was then set up for the testing of the smart hydraulic actuation system.

**Figure 7:**
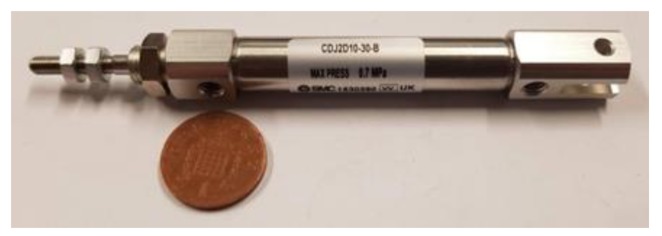
SMC CDJ2D10-30-B linear hydraulic actuator [34] shown next to a 1p coin.

In this arrangement, a Tinius Olsen tensile machine (Tinius Olsen, Horsham, PA, USA), type H5KS, was used to supply the input to the master hydraulic actuator, which was vertically aligned and clamped between the upper 500 N load cell of the machine and its lower base platform using a homemade screw-clamping mechanism. In order to simplify the experimental setup, it was decided to employ a commercially available linear hydraulic actuator, type SMC CDJ2D10-30-B (SMC Pneumatics, Yorba Linda, CA, USA) [34], shown in Figure 7, to serve as the proposed master hydraulic actuator in the developed smart actuation system.

**Figure 8:**
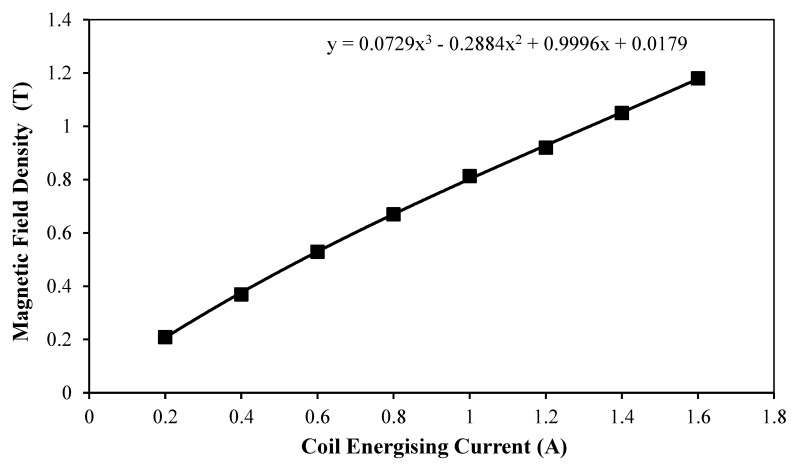
Variation of average magnetic field density simulated along the axial pole length inside the fluid gap of the MR actuator as a function of actuator’s coil-energising current.

The developed MR actuator prototype was connected to the master hydraulic actuator using PTFE tubing, and the hydraulic system was then filled with the employed MR fluid. The electrical excitation of the coil was achieved by means of a quad-mode TTi (model PL330QMT) low-voltage power supply. In the present arrangement, the master hydraulic actuator could be accurately driven through the controlled precision axial displacement input by the tensile machine, while the corresponding impeding force of the smart hydraulic actuation system under various applied excitation current levels could be measured by the load cell of the machine. In order to facilitate a realistic comparison between the theoretical and experimental results, the relationship between the magnetic flux density and the coil-energising current was estimated so that the correct fluid yield stresses could be assumed at the corresponding experimentally applied currents. Figure 8 shows the variation of the average magnetic flux densities, which were simulated along the axial pole length inside the fluid gap of the MR actuator, as a function of the coil-energising currents in the range between 0.2 and 1.6 A.

**Figure 9:**
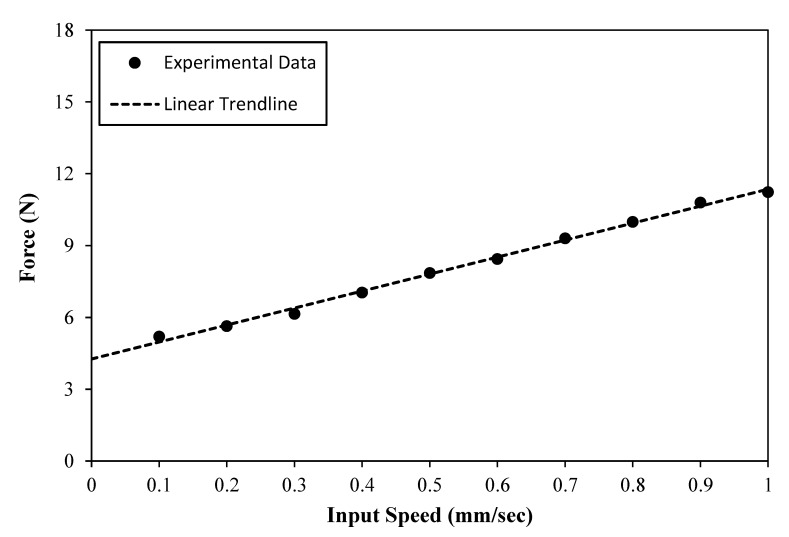
Resistance force of the smart hydraulic actuation system with deactivated MR actuator versus input speed.

The smart hydraulic actuation system was then experimentally assessed when the master hydraulic actuator was driven by the tensile machine, while the resistance force of the system was recorded using the machine’s force link for a range of input speeds, which varied between 0.1 and 1 mm/sec. This test was carried out when the MR actuator was not activated, and therefore the measured forces mainly represented the viscous and the friction forces of the system. Figure 9 shows the results of this test, which were numerically extrapolated back to zero-driving speed level to obtain the resistance force, due to the friction in the system. The zero-velocity force, which was expected to be mainly caused by the friction initiated between the mechanical seals and the shells of the master hydraulic and MR actuators, in addition to the friction inside the connecting fluid tubing, was estimated as 4.26 N.

**Figure 10:**
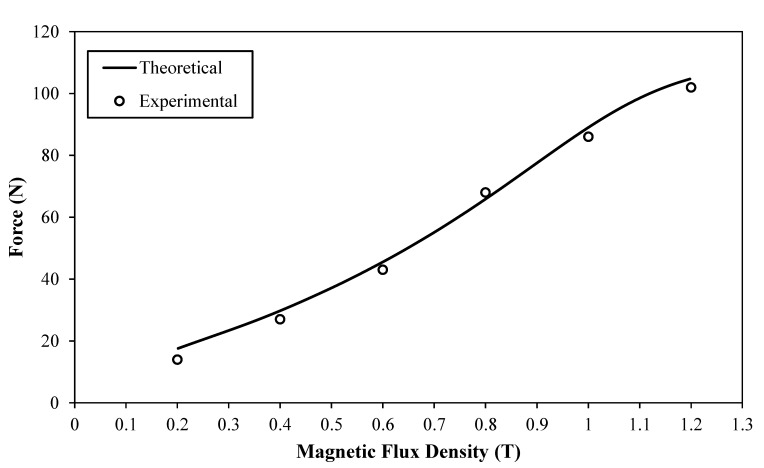
Total force of the smart hydraulic actuation system as a function of the magnetic flux density for a driving speed of 0.5 mm/s.

In this figure, however, the force seems to increase slowly with the driving speed, reaching a maximum of about 11 N at a speed of 1 mm/s. This marginal force increase beyond the friction force could be ascribed to the fact that although the effects of the speed on the flow rate of the MR fluid inside the actuators is minimal—which is why Equation (3) yields small viscous forces—this effect becomes more effective inside the connecting tubing, which results in higher viscous forces. Since the shaping of the smart SLS tool is done whilst the MR actuators are deactivated, an actuation speed of 0.5 mm/sec could complete the designed axial stroke of 6.6 mm, and produce the bending angle of 90° of the developed MR actuator in about 13 s, which is considered appropriate for a single reconfiguration of a surgical instrument used in SLS procedures. Accordingly, and in consideration of the results presented in Figure 9, a driving speed of 0.5 mm/sec would result in a resistance force of about 7.7 N when the MR actuator is not activated, which is considered to be achievable and comfortable for manual operation by the surgeon. Consequently, a speed of 0.5 mm/sec was chosen as the driving speed of the master hydraulic actuator in the measurements of the total force of the smart hydraulic actuation system, which were done for magnetic flux densities in the range between 0.2 and 1.2 Tesla (see Figure 8 for the relationship between the magnetic flux density and the coil energising current). Figure 10 shows the results of this test.

In addition, using the employed fluid properties and the MR actuator design parameters (Table 1), the magnetic field-dependent damping force (*F_τ_*) was theoretically estimated (Equation (2)) for the same input speed of 0.5 mm/s and the same magnetic flux density range (0.2 to 1.2 Tesla). These estimated forces were then added to the resistance force (*F_v_* + *F_f_*), which was measured as 7.7 N at this speed level (Figure 9), and presented in Figure 10 for a comparison with the experimentally measured total forces. It can be seen that higher magnetic flux densities, which resulted in larger fluid yield strengths, caused the smart hydraulic actuation system to produce higher total locking forces. It is worth mentioning that the employed MR fluid typically starts to show yield strength saturations for magnetic flux densities higher than 1.3 Tesla [25]. This is evidenced in Figure 10, when signs of force saturations appear for magnetic field densities at around 1.2 Tesla. However, the simulated forces in general seem to be slightly higher than those obtained experimentally. This could be attributed to the fact that although the electromagnetic circuit of the MR actuator was designed, using the electromagnetic finite element modelling technique, to generate a uniformly distributed magnetic field along the effective axial pole length inside the MR fluid gap, the prototype MR actuator may not exactly maintain this field uniformity, due to various manufacturing and experimental deviations; this would certainly affect the strength of the MR fluid, and consequently the output of the actuator. However, the developed smart hydraulic actuation system appeared to produce locking forces in excess of 100 N when the MR device was energized by a magnetic field of about 1.2 Tesla. These forces, in association with the 5 mm offset, which was allowed between the rack and pinion hinge of the top swivelling component, enabled the developed smart actuation system to resist input torques of up to 500 mNm. This torque level appears to be higher than those produced by recently reported actuating systems for SLS applications [9], and therefore the developed smart actuation system should be capable of maintaining the posture of SLS instruments against input loads that are expected under such surgical protocols. This was achieved when the developed smart actuation system, with its miniaturised MR actuator and its low excitation current feed, was capable of producing angulations of about 90°. Therefore, three of the developed MR actuators, which are embedded onto the shaft of an SLS instrument and driven by a single hydraulic actuator, could allow surgeons to experience six-degrees-of-freedom manipulations with the smart SLS tool.

## 4. Conclusions

The work reported in this paper was directed towards the design and assessment of a smart hydraulic actuation system that aimed to enhance the function of single-port laparoscopy surgical (SLS) instruments and provide them with adaptability—a role that should enable them to change their articulation without compromising patient safety. This should then improve the overall ergonomic design of SLS instruments, permitting them to access difficult angles encountered in different SLS procedures. In the developed smart system, three miniaturised magnetorheological (MR) fluid actuators are proposed to be embedded onto the shaft of conventional SLS instruments, which could be manipulated by a single hydraulic actuator with a simple manual input by the surgeon. Electromagnetic finite element simulations were applied to design the electromagnetic circuit of the MR actuator. Furthermore, the performance of the smart actuation system was assessed experimentally under controlled inputs that were provided by a tensile machine, and the results were confirmed using a theoretical approach that was based on a Bingham plastic model. The results confirmed the capability of the developed smart hydraulic actuation system to provide output forces in excess of 100 N, which could resist input torques of about 500 mNm. This output of the developed smart actuation system is compared favourably with those systems that have recently been reported for SLS applications.

## Figures and Tables

**Table 1:** The design parameters of the developed MR actuator.

MR Fluid Actuator	
Outer diameter	12 mm
Length	27.5mm
Piston Diameter, *D_p_*	9 mm
Piston Height, *L*	15 mm
Effective Axial Pole Length, *L_e_*	5 mm
Fluid Shear Gap, *h*	0.5 mm
Magnetic Material	MaxiMag Low-Carbon Magnetic Iron
Angulation Capability	0–90°
Axial Stroke	6.6 mm
